# Complete genome sequence of an astrovirus identified in a domestic rabbit (*Oryctolagus cuniculus*) with gastroenteritis

**DOI:** 10.1186/1743-422X-9-216

**Published:** 2012-09-22

**Authors:** Mark D Stenglein, Eric Velazquez, Cheryl Greenacre, Rebecca P Wilkes, J Graham Ruby, Julia S Lankton, Donald Ganem, Melissa A Kennedy, Joseph L DeRisi

**Affiliations:** 1 Departments of Medicine, Biochemistry and Biophysics, and Microbiology, University of California San Francisco, San Francisco, CA, USA; 2 Department of Small Animal Clinical Sciences, University of Tennessee College of Veterinary Medicine, Knoxville, TN, USA; 3 Department of Biomedical and Diagnostic Sciences, University of Tennessee College of Veterinary Medicine, Knoxville, TN, USA; 4 Howard Hughes Medical Institute, Chevy Chase, MD, USA

**Keywords:** Rabbit astrovirus, Rabbit virus, Virus discovery, Virochip, Gastroenteritis, Diarrhea virus, Mucoid enteropathy, Enterocolitis

## Abstract

A colony of domestic rabbits in Tennessee, USA, experienced a high-mortality (~90%) outbreak of enterocolitis. The clinical characteristics were one to six days of lethargy, bloating, and diarrhea, followed by death. Heavy intestinal coccidial load was a consistent finding as was mucoid enteropathy with cecal impaction. Preliminary analysis by electron microscopy revealed the presence of virus-like particles in the stool of one of the affected rabbits. Analysis using the Virochip, a viral detection microarray, suggested the presence of an astrovirus, and follow-up PCR and sequence determination revealed a previously uncharacterized member of that family. Metagenomic sequencing enabled the recovery of the complete viral genome, which contains the characteristic attributes of astrovirus genomes. Attempts to propagate the virus in tissue culture have yet to succeed. Although astroviruses cause gastroenteric disease in other mammals, the pathogenicity of this virus and the relationship to this outbreak remains to be determined. This study therefore defines a viral species and a potential rabbit pathogen.

## Background

The astroviruses form a family of small, non-enveloped, positive strand RNA viruses that infect a variety of mammalian and avian hosts [1-22]. First identified in human stool samples in 1975, these viruses were named after their star-shaped appearance in some electron micrographs [2,3]. Astroviruses have been shown to replicate in cells of the intestinal tracts of infected organisms, and in some hosts, infection has been demonstrated to cause gastroenteritis [6,13,23-31]. In birds, astroviruses have been linked to intestinal and extra-intestinal pathology [10,32-35].

High throughput, unbiased molecular methods can be used to identify candidate etiologic agents in diseases of unknown origin [36-38]. The Virochip is a DNA microarray that has been used to identify known and to discover novel viruses [39,40]. Next generation sequencing provides a complementary virus discovery method, and is being increasingly used as its cost decreases. The sensitivity of these methods decreases as divergence from known viruses increases and as abundance of the viral nucleic acid in the sample decreases.

In this study, the Virochip microarray was used to screen samples from an outbreak of gastroenteritis in a commercial rabbit colony in Tennessee, USA. A virus was suspected as one possible cause because of virus-like particles in electron microscopic examination of stool from an affected animal, but traditional diagnostic approaches, including virus isolation, failed to identify a candidate viral pathogen. Virochip screening suggested that an astrovirus was present, and subsequently the complete genome was recovered and characterized. Recently, the partial genome sequence of a related virus was recovered from rabbits in Italy [22]. The astroviruses form a diverse family of viruses that infect many animals, and metagenomic investigations such as the one described here will continue to reveal the full extent of their diversity and host range.

## Results

### Outbreak and clinical diagnostics

**Figure 1:**
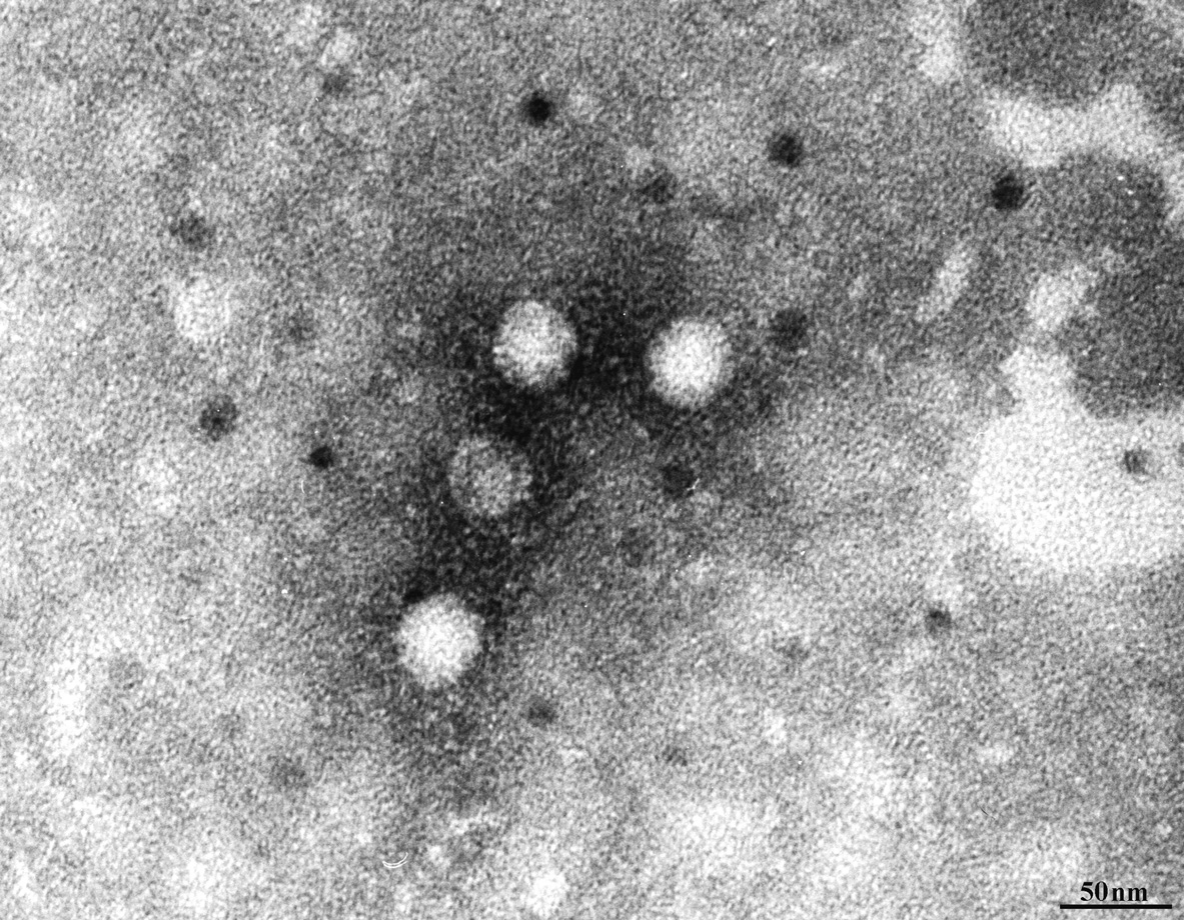
**Electron micrograph of virus like particles in the stool of one animal (Table**1**).** Scale bar indicates 50 nm.

A domestic rabbit colony of about 100 animals of various breeds near Johnson City, TN, USA, experienced an outbreak of enterocolitis. The predominant rabbit breeds were angora, satin and mini rex. No changes in diet had occurred in 1.5 years. The rabbits were housed outdoors, on the ground, in an enclosure measuring 8 x 20 feet with an attached hutch. The owner reported voles in the area. Characteristics of the outbreak included a 1–6 day history of lethargy, bloating and diarrhea, followed by death. Initially, animals under 5 months old were affected (~15 in the first 2 weeks), then the adults were affected (~75 over the next 3 weeks). Consistent clinical signs included lethargy, dehydration, increased intestinal sounds with intestinal gas (bloating) and cecal impaction with mucoid diarrhea. Severe intestinal coccidiosis (*Eimeria* spp.) was identified and the entire colony was placed on sulfamethazine in the water (Sulmet, 12.5%, Fort Dodge). Necropsy findings on seven rabbits (2 dead on arrival, 5 euthanized) were all consistent with mucoid enteropathy and cecal impaction. (Table 1) At necropsy, common gross findings within the intestinal tract included mucoid intestinal contents (5/7), cecal impaction (4/7), serosal hemorrhage (4/7) and gastrointestinal gas distention (4/7). Microscopically, common findings included lymphoplasmacytic enteritis (5/7), necrotizing heterophilic enterotyphlocolitis (2/7), intestinal coccidiosis (5/7), and gastric or cecal candidiasis (2/7). In addition to the intestinal coccidiosis identified in all animals tested, one rabbit had hepatic coccidiosis (*Eimeria stediae*), two had *Trichuris* spp., and 4 of 5 small intestinal cultures grew *E. coli* that was found not to be an attaching effacing *E. coli*. 30 nm virus-like particles were observed by electron microscopy in the stool of 1 of 2 animals tested (Figure 1 and Table 1). Follow-up PCR-based testing determined that this virus was not Rabbit Hemorrhagic Disease Virus. Initial attempts to isolate virus from 2 samples in tissue culture were unsuccessful.

**Table 1:** Summary of diagnostic findings

**Animal #**	**Enteritis**	**Died / Euthanized**	**Parasites**	**Culture**	**EM VLPs**	**Astrovirus Virochip**	**Rabbit astrovirus RT-PCR**
1	+	Died	Intestinal *Eimeria* spp.	No growth	ND	(−)	(−)
2	+	Euthanized	*Trichuris* spp.	*E. coli*	ND	(−)	(−)
				*C. perfringens*			
3	+	Died	Intestinal *Eimeria* spp.	ND	ND	ND	ND
			Hepatic *Eimeria* spp.				
4	+	Euthanized	Intestinal *Eimeria* spp.	*E. coli*	(−)	(−)	(−)
5	+	Euthanized	Intestinal *Eimeria* spp.	*E. coli*	ND	ND	ND
6	+	Euthanized	Intestinal *Eimeria* spp.	*E. coli*	(+)	(+)	(+)
7	+	Euthanized	Intestinal *Eimeria* spp.	ND	ND	ND	ND
			*Trichuris* spp.				
8 (Control)	–	ND	ND	ND	ND	(−)	(−)

EM VLPs, virus-like particles evident by electron microscopic examination. ND, not determined. *E. coli*, *Escherichia coli*. *C. perfringens*, *Clostridium perfringens*.

### Genome recovery and phylogenetic analysis

**Figure 2:**
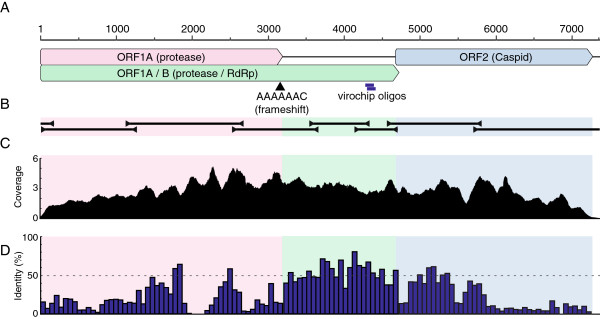
**The rabbit astrovirus genome.** (**A**) A cartoon showing the organization of the viral genome. Scale bar indicates nucleotide position. The location of a putative ribosomal frameshift motif is indicated by an arrowhead. The position of the two overlapping Virochip probes that were originally positive for an astrovirus are indicated. (**B**) Location of overlapping PCR, 5^′^, and 3^′^ RACE amplicons used to determine and confirm the sequence of the viral genome. (**C**) A histogram showing the number of Ion Torrent reads (x1000 reads) aligning at each nucleotide position in the genome. (**D**) The average pairwise percent identity between the predicted rabbit astrovirus protein sequences and the sequences of the proteins from the astroviruses shown in Figure 3. The histogram is binned into 20 amino acid windows.

Because virus-like particles were observed but diagnostic testing failed to identify known viruses, several samples were subjected to Virochip analysis. Total nucleic acid was extracted from the stool of four sick animals and one unaffected control, fluorescently labeled, and hybridized to the Virochip (other case samples lacked sufficient remaining material). To enrich for viral nucleic acid, the stool sample from the EM virus-positive sample was filtered and treated with micrococcal nuclease, to digest non-protected nucleic acid. Virochip analysis of the nuclease-treated sample suggested the presence of astrovirus-related sequence (Table 1 and Figure 2).

To validate this finding, several sets of degenerate primers were designed based on the positive Virochip probe sequences and on conserved regions of astrovirus genomes (Figure 2 and Table 2). PCR using one of these primer pairs produced an amplicon of the expected size, which was cloned and sequenced, confirming that nucleic acid from an apparently divergent astrovirus was present in the sample. Additional degenerate primers and 3^′^ RACE were used to recover a 5 kbp contiguous sequence. But, repeated attempts using 5^′^ RACE and other PCR-based strategies failed to recover the full predicted ~7 kb genome.

**Table 2:** Oligonucleotides used in this study

**Oligo name**	**Note**	**Sequence (5**^**′**^**– 3**^**′**^**)**
MDS-4	Reverse transcription	CGCTCTTCCGATCTNNNNNN
MDS-189	Library amplification	CTGTCTGGCTCTTCCGATCT
MDS-115	Degenerate astrovirus consensus	CCATCAGGTCARWWNTCAACAAC
MDS-116	Degenerate astrovirus consensus	CTCGCTAATHTAYGGDGATGA
MDS-117	Degenerate astrovirus consensus	GGTTTNACCCACATNCCAAA
MDS-118	Degenerate astrovirus consensus	GTCCAACTGTGADNCCACARAA
MDS-119	Rabbit astrovirus diagnostic	ATAATAACATGGTCAACTATTGGCTTC
MDS-120	Rabbit astrovirus diagnostic	GACATCCTTATACATTTTCACAACTTT
MDS-156	Vertebrate rRNA (L2513)	GCCTGTTTACCAAAAACATCAC
MDS-157	Vertebrate rRNA (H2714)	CTCCATAGGGTCTTCTCGTCTT
MDS-298	Genome recovery and sequencing (27–46 forward)	CGGCCAGAGAGCCTATTACC
MDS-296	5^′^ RACE (149–168 reverse)	GGCTAGAGGAATGGGGTCAG
MDS-299	Genome recovery and sequencing (1146–1165 forward)	GCTCGTTCCGATAATTGCTC
MDS-300	Genome recovery and sequencing (1229–1248 reverse)	GCAACTAACCACGCACAATG
MDS-144	Genome recovery and sequencing (2554–2564 forward)	TCTGGACYGARGARGARTA
MDS-300	Genome recovery and sequencing (2639–2659 reverse)	CACTCCATTCAGGGTAACCAA
MDS-135	Genome recovery and sequencing (3573–3592 forward)	TCCACTCCCGCCTACCCCAA
MDS-145	Genome recovery and sequencing (3622–3641 reverse)	CCACCCACAATCAGAGAGGT
MDS-119	Genome recovery and sequencing (4165–4191 forward)	ATAATAACATGGTCAACTATTGGCTTC
MDS-125	Genome recovery and sequencing (4287–4308 reverse)	CATAATCAGATGGGAGGACAGG
MDS-147	Genome recovery and sequencing (4602–4620 forward)	CTTCGCAGCCACTCTCTTG
MDS-137	Genome recovery and sequencing (4668–4683 reverse)	TTGGTCCTCCCCTCCA
MDS-138	3^′^ RACE (5719–5729 forward)	TGGTGGTTTGT
MDS-150	Genome recovery and sequencing (5759–5779 reverse)	TCTGAAATTGCACTGTGTTGG
MDS-121	RACE oligo-dT primer	CCAGTGAGCAGAGTGACGAGGACTCGAGCTCAAGCT_17_
MDS-122	RACE outer adapter primer	CCAGTGAGCAGAGTGACG
MDS-123	RACE inner adapter primer	GAGGACTCGAGCTCAAGC

To recover the remainder of the genome, metagenomic sequencing using the Ion Torrent PGM instrument was performed [41]. This produced 3x10^6^ sequences of median length 125 nucleotides (standard deviation 17.5). The average Sanger quality score of these sequences was 19.5 at base 1, 17.3 at base 100, and 14.4 at base 150, quality values consistent with previously reported metrics for this sequencing platform [42]. Insertions and deletions, which occurred at a frequency of 1.3% (1.3 indels per 100 bases aligned), were more frequent than mismatches, which occurred at a frequency of 0.28%. Retrospective mapping of the sequences revealed that 1.9×10^5^ unique sequences (6% of sequences) derived from the astrovirus, corresponding to >1000x average coverage of its genome (Figure 2). The PRICE de novo targeted assembly software (Graham Ruby, http://derisilab.ucsf.edu/software/price/) was used to assemble the entire viral genome, the sequence of which was validated by RT-PCR and Sanger sequencing (Figure 2). 5^′^ RACE was used to confirm that the Ion Torrent-based assembly extended to the 5^′^ terminus of the genome (Figure 2). The remainder of the reads in the metagenomic dataset consisted mainly of sequences derived from bacteria (~76% of taxonomically assigned reads), with the *Escherichia*, *Akkermansia*, *Clostridium*, *Salmonella*, *Enterobacter*, *and Shigella* genera being most prevalent. 56 sequences (0.002% of all sequences) aligned best to Eimeria species, consistent with the clinical diagnosis of coccidiosis (Table 1). A small percentage (0.4%) of human sequences were also evident in the data. These were likely laboratory contaminants, as the majority of them aligned to the breast cancer 1 and 2, early onset genes (BRCA1 and BRCA2), and targeted sequencing of these genes was conducted in the laboratory where the sequencing libraries were prepared. Library contamination was also the likely source of 27 reads with nucleotide sequences nearly identical to human immunodeficiency virus-1 (HIV-1) database sequences.

In an attempt to propagate and isolate the virus, stool from the positive sample was filtered and used to inoculate a rabbit kidney cell line (RK) culture and cultures of several other mammalian cell lines previously shown to support the replication of human astroviruses: Vero, HT-29, and Caco-2 [43-45]. These cultures were maintained for 15 days, and culture supernatants were collected on days 1, 2, 4, 6, 10, and 15 post innoculation. Rabbit astrovirus RNA was detected in the day 1 supernatant (in the innoculum) by RT-PCR but not in later time points, suggesting that the virus did not replicate in these cultures.

**Figure 3:**
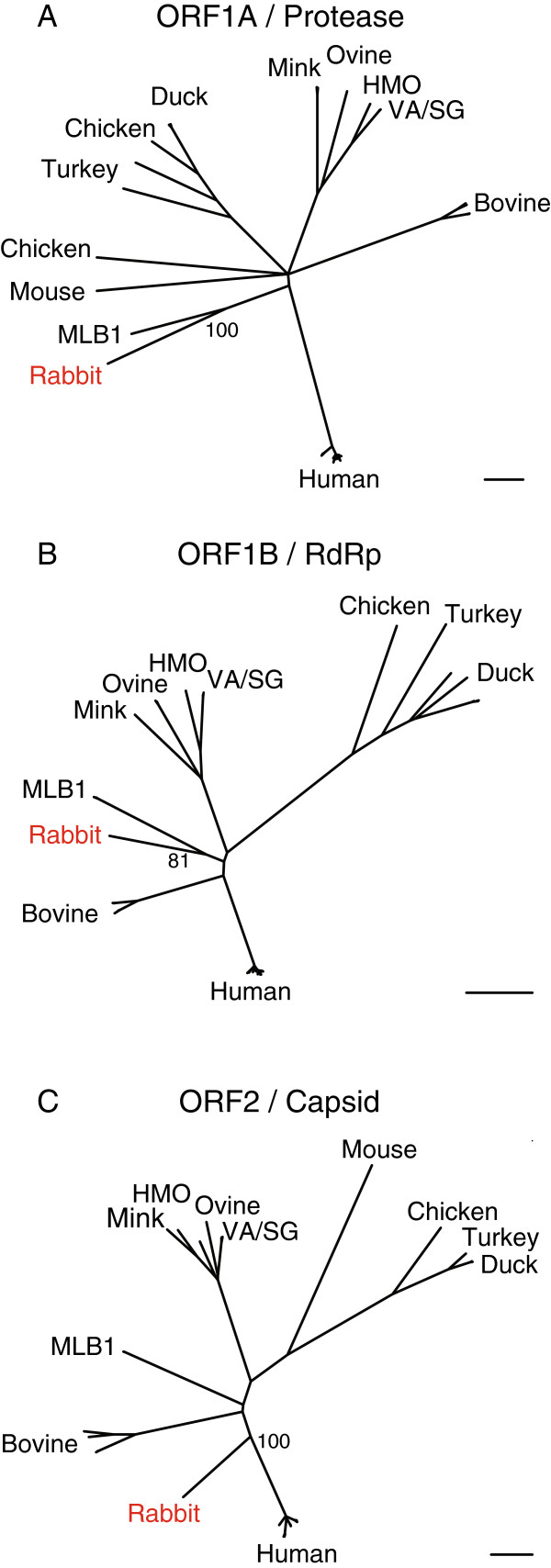
**Maximum likelihood phylogenies created from multiple sequence alignments of the predicted rabbit astrovirus proteins and the related proteins of all astroviruses in Genbank with complete genome sequences.** (**A**) Phylogeny based on ORF1A sequences. (**B**) Phylogeny based on ORF1B sequences. (**C**) Phylogeny based on the first ~420 amino acids of the indicated ORF2 (capsid) sequences. See Methods for accession numbers of sequences used. The bootstrap percentages of selected nodes are indicated. Scalebars indicate 0.2 amino acid substitutions per site.

A phylogenetic analysis of the predicted rabbit astrovirus protein sequences was performed. The rabbit astrovirus sequences were compared to those of all the astroviruses in GenBank for which a complete genome sequence exists (see Methods). Analysis with the three major viral polyproteins, nsp1A, nsp1B (RdRp), and capsid (see Discussion) produced overall similar tree topologies (Figure 3). The rabbit astrovirus sequences branch basally on the phylogram, with the closest related sequences being those of Astrovirus MLB1, which was isolated from a human [46]. The rabbit virus nsp1A, nsp1B, and capsid proteins share 30%, 59%, and 25% pairwise amino acid identity with the corresponding Astrovirus MLB1 protein sequences. In the phylogenies based on nsp1A and nsp1B, the clade formed by the rabbit virus and Astrovirus MLB1 is supported by 100% and 81% of boostrap replicates, respectively (Figure 3A and B). In contrast, the rabbit virus capsid sequence does not form a well-supported clade with the astrovirus MLB1 capsid sequences, but instead branches along the lineage leading to the canonical human astroviruses.

Diagnostic primers were designed to specifically detect rabbit astrovirus (MDS-119 and −120; Table 2). RT-PCR using these primers indicated that, of the five stool samples collected (from four sick and one control rabbit), only the EM-positive sick rabbit tested positive for the virus (Table 1).

## Discussion

In this study, we describe the identification and genomic characterization of a member of the *Astroviridae* family of viruses. Traditionally, astroviruses have been named after the host species from which they were identified, so we propose naming this virus species *Astrovirus rabbit/TN/2009/USA*.

This virus was isolated from the feces of a rabbit suffering from enterocolitis, but it is not clear that the virus caused the disease in this instance. Indeed, samples from three of the other four sick rabbit from the same outbreak tested negative for the virus (Table 1). Nevertheless, astroviruses cause gastroenteritis in other mammals, and it is possible that this virus caused or exacerbated the illness in a subset of the animals [13,24,25,28,29,47]. The coccidian parasites and bacteria evident in the samples may have also contributed to the enteritis and from this data alone, it is impossible to ascertain whether this astrovirus is capable of causing disease. Unfortunately, the sample material was limited, and attempts to propagate the virus in culture were unsuccessful, so experiments to directly demonstrate disease causality were not possible. It may be possible to generate an infectious clone of the virus, as has been accomplished for other astrovirus species [48]. Screening of additional rabbits with diarrhea will provide additional epidemiological information and another source of virus for follow-up experiments.

Indeed, as this manuscript was being prepared, the partial sequence of an astrovirus recovered from rabbits in Italy was reported [22]. In this report, viral RNA was detected by RT-PCR in 10 of 23 rabbits (43%) with enteritis and in 18% of 139 asymptomatic animals, and the mean copy number was ~100-fold higher in the feces of the sick rabbits compared to the apparently healthy animals. The 3395 nt sequence (accession JN052023) in the Martella *et al.* report includes a portion of ORF1B and ORF2, and is 92% identical at the nucleotide level to the sequence recovered from the Tennessee rabbit. Thus, similar astroviruses appear to be a common and geographically widespread infection of rabbits, and an increased viral load is associated with enteritis.

Astroviruses were so named because of their star-shaped appearance in electron micrographs [3]. Although non-enveloped virus-like particles of a size typical of astroviruses (~30 nm) were observed in the astrovirus RNA-positive sample, it is not clear that these particles correspond to the astrovirus. It is important to note that as few as 10% of particles in a preparation may exhibit the characteristic star-shaped morphology [3].

The rabbit astrovirus genome contains characteristics typical of astrovirus genomes, and it is therefore possible to make predictions about viral gene expression and function [1]. Like other astroviruses, the rabbit astrovirus genome is about 7 kb in length and contains three large open reading frames, named ORF1A, ORF1B, and ORF2. It is likely that ORF1A can be translated by itself or in conjunction with ORF1B via a −1 ribosomal framseshift at a conserved AAAAAAC motif (Figure 2) [49-51]. ORF1A and ORF1AB encode the viral non structural polyproteins nsp1A and nsp1ab. ORF2 encodes the viral structural capsid polyprotein. These polyproteins are predicted to be proteolytically processed into multiple domains with discrete functions [52-57].

Astrovirus nsp1A proteins contain protease domains, but apart from this their function is not well characterized. According to NCBI’s Conserved Domain Database search tool (CDD) the predicted rabbit astrovirus nsp1a protein contains two conserved domains: a serine protease motif, and a chromosome segregation protein DNA-binding domain [58]. This DNA binding-related domain may bind RNA and be involved in viral replication. As has been reported for other astroviruses, the nsp1A protein is predicted to contain several transmembrane and coiled coil domains [59]. It is likely that the nsp1A contains other functional domains of as yet unknown function. rabbit astrovirus nsp1A contains a 76 amino acid insertion between residues 674 and 749 not found in related protein sequences. These residues contain no obvious identifiable domain.

ORF1B is predicted to encode nsp1B, which results from proteolytic cleavage of nsp1ab. Nsp1b is the RNA-dependent RNA polymerase (RdRp), and this is the only domain identifiable by CDD or BLAST search. The nsp1B protein is the most conserved of the three polyproteins (Figure 2).

ORF2 encodes the viral structural (capsid) precursor polyprotein. As is the case with other astroviruses, the structural polyprotein may be translated from a subgenomic RNA, although no evidence of such a species is evident in the deep sequencing coverage levels (Figure 2) [60,61]. It is possible that the subgenomic RNA is present is at higher levels in infected cells than in extracellular virus particles, which are likely the source of the filtered material sequenced. The region defined by the first 70 amino acids of the ORF2-encoded protein is basic, with a predicted isoelectric point of 13.3. This is consistent with related astrovirus proteins, in which this domain has been reported to be involved in nucleic acid binding [55]. The N-terminal ~450 amino acids of the capsid protein align by BLAST with other astrovirus capsid proteins, but no recognizable protein domains are contained in the C-terminal 400 amino acids, and the latter half of the protein exhibits a much lower degree of sequence similarity to other astrovirus proteins sequences (Figure 2). This is in agreement with the model that the N-terminal half of astrovirus capsid polyproteins form a conserved core domain and the C-terminal halves of the proteins are highly variable receptor interacting domains [62]. ORF1A is preceeded by a 12 nt untranslated region, which is short but within the size range (11–85 nt) previously reported for astroviruses [1]. The 87 nt predicted 3^′^ untranslated region is followed by a polyA tail.

## Conclusions

Here, we identify and fully characterize the genome of an astrovirus present in the stool of a rabbit suffering from a fatal intestinal disease. This was enabled by the combination of two complementary genomics techniques: the Virochip microarray and high throughput sequencing on the Ion Torrent PGM platform. Although the precise contribution of this virus to the observed pathology remains to be determined, related viruses cause similar disease in other mammals, and this virus is a plausible etiologic agent. The molecular information presented here sets the stage for confirmatory follow-up studies.

## Methods

### Clinical methods

Fresh fecal samples were submitted to the Clinical Virology Laboratory (University of Tennessee Veterinary Medical Center) for electronmicroscopic examination. Samples were processed as described in [63]. Briefly, samples were centrifuged in distilled water at approximately 15,000×g for one hour. The supernatant was discarded and pellet resuspended in 2–3 ml distilled water. 100ul of the resuspended material was added to 1 ml distilled water containing 3% phosphotungstic acid. The solution was aerosolized onto a carbonate-coated copper grid and examined by EM at 30,000 and 300,000X.

### Nucleic acid extraction

Total nucleic acid was extracted from stool samples using the Qiagen QiaAmp DNA mini kit, which purifies RNA and DNA, according to the manufacturer’s protocol. Additionally, the EM VLP-positive stool sample (sample ID 10–2208) was diluted in PBS, clarified by centrifugation at 10,000 g for 1 minute, and passed through a 0.22 um filter (Millipore). The Filtrate was treated with microccoal nuclease (MNase; NEB) in order to degrade non-encapsidated (or otherwise not protected) nucleic acid. MNase reactions containing 1x reaction buffer, 100 μg/ml BSA, and 4, 1, 0.2, or 0 units MNase were incubated at room temperature for 15 minutes. MNase was inactivated by addition of EGTA to 20 mM.

### Library preparation

For virochip analysis, randomly-primed libraries were prepared from total nucleic acid. RNA was reverse transcribed in reactions containing 1x reaction buffer, 5 mM dithiothreitol, 1.25 mM dNTPs, 20 pmoles primer MDS-4 (Table 2), 100 U Superscript III (Invitrogen), and 100 ng template. Following reverse transcription, Sequenase reaction buffer and 2 U of Sequenase DNA polymerase (Affymetrix) were added to samples for second strand synthesis. The Sequenase reactions were performed twice so that starting DNA templates would be converted into end-tagged library molecules. The resulting libraries were amplified by PCR using primer MDS-189 (Table 2). PCRs contained 1x reaction buffer, 2 μM primer, 0.25 mM dNTPs, 2 U taq DNA polymerase, and 2 μl library template. Thermocycling was: 95°C for 2 min; 2 cycles of 95°C for 30 sec, 40°C for 30 sec, and 72°C for 1 min, then 25 cycles of 95°C for 30 sec, 58°C for 30 sec, and 72°C for 1 min, with a final extension of 72°C for 5 min.

For microarray hybridzation, a fraction of each library was amplified by PCR as above but with a modified dNTP mixture including 5-(3-aminoallyl)-dUTP (Ambion) in lieu of 75% of the dTTP normally in the mixture. The resulting amino-allyl-containing DNA was purified using a DNA Clean and Concentrator-5 column (DNA-CC-5; Zymo Research). The eluate was heat denatured at 95°C for 2 min, cooled briefly on ice, then fluorescently labeled in reactions containing 100 mM sodium bicarbonate pH 9, 10% DMSO, and 667 μM Cy3 mono NHS ester (GE Healthcare) for 1 hour at 25 °C. Labeled DNA was purified using the DNA-CC-5 columns and added to hybridization reactions containing 3xSSC, 25 mM HEPES pH 7.4, and 0.25% SDS. Hybridization mixtures were heated at 95°C for 2 minutes then applied to microarrays and hybridized overnight at 65 °C. Following hybridization, arrays were washed twice in 0.57xSSC and 0.028% SDS and twice in 0.057x SSC, then scanned on an Axon GenePix 4000B microarray scanner. Three tools were used to analyze Virochip data: E-predict [32], Z-score analysis [33], and cluster analysis [34].

### PCR/Sanger sequencing

Oligonucleotide sequences used to amplify the rabbit astrovirus genome are listed in Table 2. PCRs contained 1x reaction buffer, 2 μM primer, 0.25 mM dNTPs, 2 U Taq DNA polymerase, and 2 μl library template. Thermocycling was: 95°C for 2 min, then 30 cycles of 95°C for 30 sec, 58°C for 30 sec, and 72°C for 2 min, with a final extension of 72°C for 5 min. Amplicons were purified from agarose gels using the PureLink gel extraction kit (Invitrogen) and cloned into the pCR2.1 TOPO vector (Invitrogen) according to the manufacturer’s protocols. Cloned amplicons were sequenced on an ABI 3700 instrument. For diagnostic testing, primers MDS-119 and −120 (Table 1) were used with reaction and thermocycling as described above.

### 5^′^ and 3^′^ RACE

5^′^ and 3^′^ RACE were performed essentially as described [64,65], with primers listed in Table 2. RACE amplicons were cloned and sequenced as described above.

### Ion Torrent metagenomic sequencing

Randomly primed cDNA was prepared in reverse transcription reactions containing 1x reaction buffer, 5 mM dithiothreitol, 1.25 mM dNTPs, 20 pmoles random hexamer primer, 100 U Superscript III (Invitrogen), and 100 ng RNA template. Second strand DNA was synthesized as above using Sequenase DNA polymerase. cDNA was adapted for Ion Torrent sequencing using the Ion Xpress Library Kit (catalog # 4468987) according to the manufacturer’s protocols. Although this protocol includes PCR amplification, identical reads were removed during analysis to eliminate possible PCR duplicates. Sequencing was performed on an Ion Personal Genome Machine on one model 314 chip and one model 316 chip according to the manufacturer’s protocols.

### Tissue culture

100 μl of stool positive for rabbit astrovirus RNA by RT-PCR was diluted 1:10 in PBS and filtered through a 0.22 μm filter (Millipore). Filtrate was used to inoculate cultures of RK, Vero, HT-29, or Caco-2 cells, which were grown in DMEM supplemented with 10% fetal bovine serum and 50 units/ml penicillin and 50 μg/ml streptomycin at 37°C and 5% CO2. Cultures were supplemented with 0, 1, 10, or 100 μg/ml trypsin, which has been shown to be necessary for astrovirus infectivity in tissue culture. Culture supernatant was harvested and replaced at the indicated time points and stored at −80°C until processing. RNA was extracted from supernatant and reverse transcribed as described above. PCR using primers MDS-119 and −120 for rabbit astrovirus and MDS-156 and −157 for rabbit rRNA (as a positive RT-PCR control) was used to detect viral RNA, with reaction conditions as described above (See Table 2 and [66]).

### Bioinformatics

Astrovirus protein sequences for all astroviruses with complete genome sequences were downloaded from GenBank. The accessions of these sequences are: AB308374, AF260508, AY179509, AY720891, AY720892, DQ028633, DQ070852, DQ344027, EU111742, FJ222451, FJ402983, FJ434664, FJ755402, FJ755403, FJ755404, FJ755405, FJ919225, FJ919226, FJ919227, FJ919228, FJ973620, GQ415660, GQ495608, GQ891990, GU985458, HM237363, HM450380, HQ398856, HQ916313, HQ916314, HQ916316, HQ916317, JF414802, JF755422, L23513, NC_001943, NC_002469, NC_002470, NC_003790, NC_004579, NC_005790, NC_010646, NC_011400, NC_012437, NC_013060, NC_013443, NC_014320, NC_015935, and Y15937. Sequences were aligned using Clustal (version 2.0.12) using default parameters [67]. Alignments were manually inspected and trimmed to the point of clear homology. The ORF2 (capsid) alignment is based on the first ~420 amino acids of the protein sequences, which correspond to the relatively conserved core region of the capsid protein. Neighbor joining trees were constructed using PhyML software (PhyML plugin for Geneious version 2.0.12) using 100 bootstrap replicates and default parameters [68]. The sequence of the rabbit astrovirus has been deposited in GenBank, with accession JF729316.

The BLAST alignment software (version 2.2.25+) was used to taxonomically categorize the sequences in the Ion Torrent dataset [69]. The NCBI non-redundant nucleotide database was searched using the blastn algorithm with an expect value cutoff of 1e-6. For each query producing an alignment, the taxonomic ID of the best alignment was determined and tallied. The Paired-Read Iterative Contig Extenion (PRICE, version 0.13) *de novo* assembler (freely available at: http://derisilab.ucsf.edu/software/price/index.html) was used to assemble the rabbit astrovirus genome from the Ion Torrent dataset.

Several tools were combined to determine coverage, quality, and error metrics. First, CD-HIT was used to collapse identical sequences (cd-hit-est version 4.5.4 run with parameter –c 1), which may be PCR duplicates [70]. Then, the bowtie2 aligner was used to map unique reads to the Sanger-verified viral genome assembly (version 2.0.0 run in –local mode with otherwise default parameters; [71]). The number of gap openings (XO field) and mismatches (XM field) and the total number of aligned bases in the bowtie2 SAM output were tallied to determine the frequency of indels and mismatches. Average per base quality scores were determined directly from the Ion Torrent FASTQ output.

## Competing interests

The authors declare no competing interests.

## Authors’ contributions

MDS performed library preparation, microarray analyses, genome sequencing, bioinformatic analyses, and drafted the manuscript. EV performed library preparation and microarray analyses. CG extracted nucleic acid and conducted clinical examinations and analyses. RW performed electron microsopy and nucleic acid extraction and clinical diagnostics. JGR wrote the PRICE software and assisted in bioinformatic analyses. JSL performed pathologic examination and analyses. DG, MAK, and JLD oversaw project design and coordination. All authors read and approved the final manuscript.
